# Anaesthetic management for awake craniotomy in brain glioma resection: initial experience in Military Hospital Mohamed V of Rabat

**DOI:** 10.11604/pamj.2017.27.156.10249

**Published:** 2017-06-30

**Authors:** Mohammed Meziane, Abdelghafour Elkoundi, Redouane Ahtil, Miloudi Guazaz, Bensghir Mustapha, Charki Haimeur

**Affiliations:** 1 Department of Anesthesiology and Intensive Care, Military Hospital Med V of Rabat, Faculty of Medicine and Pharmacy of Rabat, University Souissi-Med V, Rabat, Morocco; 2 Department of Neurosurgery, Military Hospital Med V of Rabat, Faculty of Medicine and Pharmacy of Rabat, University Souissi-Med V, Rabat, Morocco

**Keywords:** Awake craniotomy, brain glioma resection, control target infusion, laryngeal mask airway, scalp nerve block

## Abstract

The awake brain surgery is an innovative approach in the treatment of tumors in the functional areas of the brain. There are various anesthetic techniques for awake craniotomy (AC), including asleep-awake-asleep technique, monitored anesthesia care, and the recent introduced awake-awake-awake method. We describe our first experience with anesthetic management for awake craniotomy, which was a combination of these techniques with scalp nerve block, and propofol/rémifentanil target controlled infusion. A 28-year-oldmale underwent an awake craniotomy for brain glioma resection. The scalp nerve block was performed and a low sedative state was maintained until removal of bone flap. During brain glioma resection, the patient awake state was maintained without any complications. Once, the tumorectomy was completed, the level of anesthesia was deepened and a laryngeal mask airway was inserted. A well psychological preparation, a reasonable choice of anesthetic techniques and agents, and continuous team communication were some of the key challenges for successful outcome in our patient.

## Introduction

The awake brain surgery is an innovative approach in the treatment of tumors in the functional areas of the brain, which is common for glioma [1]. This technique has multiple objectives: to maximize the extent of tumor resection but also to limit the neurological damage [2]. The anesthetic goals are to maintain an awake and cooperative patient during cortical mapping stage and ensure the safety of the airway, an adequate cerebral perfusion and brain relaxation [3].

## Patient and observation

A 28-year-oldNorthAfrican male underwent an awake craniotomy for brain glioma resection. The patient was diagnosed with glioma in the left temporal lobe from transient episodes of confusion dating back to a month ago. At the preanesthetic visit, the neurological examination was normal. The airway examination did not indicate any predicted difficulties for tracheal intubation or mask ventilation. The rest of his physical examination revealed no cardiac or respiratory abnormalities and the patient was classified ASA 1. The preoperative values of serum electrolytes and blood count were normal. Both of the neurosurgical and anesthetic team have explained the entire surgical procedure to the patient with emphasis on the awake phase. After accepting the anesthetic protocol, the informed consent form was signed. No premedication was prescribed. In the operating room, the patient was calm, well oriented with GCS of 15/15. EKG, non-invasive blood pressure, and the pulse oximetry were monitored. Two intravenous lines were placed (16 and 18 G). Prophylactic antibiotic therapy (cefazolin 2 g), ondansetron (8 mg) and dexamethasone (8 mg) were administered. The scalp nerve block was performed with Bupivacaine 0.5% and Lidocaine 2%; 3 ml of local anesthetics were injected in the supraorbital, supratrochlear, auriculotemporal, lesser occipital and greater occipital nerves. After that, the neurosurgeon infiltrates the pin sites with lidocaine 2% before installing the Mayfield head fixation device. Sedation with control target infusion with propofol and remifentanil was started. A low sedative state (Ramsey score: 2) was maintained with propofol concentration at 1 µg/ml and remifentanil concentration at 1 ng/ml. A simple oxygen face mask was placed, with 6 L/min of oxygen. Carbon dioxide sampling line was placed in the right nostril, and the patient's breathing was monitored. An arterial line was also placed in the right radial artery and a urinary catheter was inserted for patient comfort during the operation. The patient was then placed in the right lateral position. To avoid fatigue and discomfort, shoulders, arms, and lower extremities were supported by pillows. At the time of scalp incision and until removal of bone flap, the concentration of propofol and remifentanil was respectively increased to 4 µg/ml and 3 ng/ml.Mannitol0.75 g/kg IV, was administered to facilitate surgical exposure of the brain. Blood pressure after skin incision and during craniotomy remained stable and the patient did not complain any pain. During brain glioma resection, the patient awake state was maintained without any complications. He was able to move his fingers and limbs and was continuously made to answer questions. During surgery, the systolic blood pressure was maintained at 110-140 mmHg, respiratory rate at 10-20/min and ETCO2 30-35 mmHg. Oxygen saturations throughout the entire case were 100%. Once, the tumorectomy was achieved, the level of anesthesia was deepened and a laryngeal mask airway (LMA) was inserted. After skin closure and removal of the Mayfield fixation, propofol and remifentanil infusions were stopped. Paracetamol 1 g and ketoprofen 100 mg were administered as analgesia. The patient was awakened and the LMA was removed in the operating room. The patient's postoperative course was uncomplicated and neurologic examination remained normal.

**Informed consent:** written informed consent was obtained from patient who is described in this case report.

## Discussion

We report our first experience on anesthetic management for awake craniotomy for brain glioma resection. This technique is designed to enable a tailored resection and to maximize the tumor removal with a few side effects on the neurological function [2] especially for tumors that arein close proximity to the eloquent areas, which is common for gliomas [1]. The current supporting evidence shows that awake craniotomy is associated with better outcome, earlier hospital discharges and fewer neurological deficit [3, 4]. A recent cohort study, involving 575 patients, has compared the gross total resection rate and postoperative neurological deficits between awake craniotomy and general anesthesia. AC was associated with a higher gross total resection rate in the eloquent area and fewer postoperative neurological deficits [5]. There are various anesthetic techniques for AC, including asleep-awake-asleep (SAS) technique, monitored anesthesia care (MAC), and the recent introduced awake-awake-awake (AAA) method. SAS is the oldest technique using general anesthesia with the use of a laryngeal mask airway or endotracheal intubation during the positioning, head-spinning and craniotomy, and put back for the skin closure. The general anesthesia is discontinued during the brain mapping. MAC consists of conscious sedation, where the sedatives and analgesics are titrated based on the surgical stages while the patient is maintained with spontaneous breathing and able to follow orders. The combination of propofol and remifentanil infusion is commonly used. Both of them have short half-lives and are easy to titrate. In addition, Propofol decreases the cerebral metabolic rate, and has anticonvulsant and antiemetic effects. Dexmedetomidine has gained popularity for use in awake craniotomy owing to its minimal respiratory depressant effects. However, there have been reports of wide variability of clinical outcomes [6–8]. AAA only consists of local or regional anesthesia with intravenous analgesia but avoiding sedative anesthetics.

One of the recent systematic reviews and metanalysis, involving 47 studies and comprising 5945 AC, has analyzed the recent evidence of benefits and harms resulting from the different anesthesia techniques of AC. SAS and MAC for AC seem to be similarly safe without serious complications, whereas evidence for the AAA technique is limited [9]. In our case, we used a combination of these techniques. A local anesthesia with scalp nerve bloc and sedation with spontaneous breathing were performed until the cortical mapping stage. At this point, the patient was awakened. When the tumorectomy was finished, a general anesthesia was induced and the surgery was then completed. This approach is convenient because it allowed us to maintain the awake state only the period the patient must be cooperative and provided a deeper level of anesthesia for the patient during the cranial and scalp suturing which is the most painful and stimulating part of the surgery. The addition of a local anesthesia with scalp nerve block was used to improve the analgesic quality and reducethe hypnotic requirement and in consequence, the respiratory depressant effect. AC poses unique challenges, especially in management of unprotected airway and limited access to the patient as the head is fixated to the Mayfield head immobilizer and due to positioning. The medical staff should always be ready to switch to total anesthesia. The most commonly occurring accidents are seizures, agitation, apnea, vomiting, nausea, neurological deficit, respiratory depression, air embolisms, and loss of patient cooperation. In the presented case, no intraoperative complications occurred. The patient was premedicated with ondansetron and dexamethasone, so he did not experience nausea or vomiting. Mannitol was used in order to prevent brain tightness. The patient may refuse to cooperate for different reasons, including poor preoperative preparation, inappropriate sedation, insufficient analgesia, uncomfortable position or prolonged surgical time. In our case, a well psychological preparation, a reasonable choice of anesthetic techniques and agents, and continuous team communication were some of the key challenges for successful outcome in our patient.

## Conclusion

Awake craniotomie are advantageous and becoming more popular practice that allows for increased tumor resection and decreased postoperative neurologic morbidity. This technique, however, presents many challenges to both the neurosurgeon and anesthetist. We conclude that anesthesia with scalp nerve block, propofol-remifentanil target controlled infusion combined with the use of the LMA after removal of the tumor may be an alternative anesthetic technique for awake craniotomies.

## Competing interests

The authors declare no competing interests.
